# Endodontic Outcome of Root Canal Treatment Using Different Obturation Techniques: A Clinical Study

**DOI:** 10.3390/dj11080200

**Published:** 2023-08-21

**Authors:** Alexander Winkler, Philipp Adler, Julia Ludwig, Norbert Hofmann, Sebastian Soliman, Gabriel Krastl, Ralf Krug

**Affiliations:** 1 Center of Dental Traumatology, Department of Conservative Dentistry and Periodontology, University Hospital Würzburg (UKW), Pleicherwall 2, 97070 Würzburg, Germany; ludwig_j4@ukw.de (J.L.); hofmann_n1@ukw.de (N.H.); soliman_s@ukw.de (S.S.); krastl_g@ukw.de (G.K.); krug_r@ukw.de (R.K.); 2 Department of Cranio-Maxillofacial Surgery, University Hospital Münster, Albert-Schweitzer-Campus 1, Gebäude W 30, 48149 Münster, Germany; philipp.adler@ukmuenster.de

**Keywords:** endodontic outcome, root filling, adhesive obturation, Resilon, GuttaFlow

## Abstract

Objective: To evaluate the clinical outcome of root canal treatment by obturation technique, root canal filling quality, and tooth- and patient-related variables. Methods: This clinical study investigated the endodontic outcome of 114 teeth treated between the years 2009 and 2012. Three different obturation methods were used: (1) adhesive obturation using the continuous wave of condensation technique with Resilon^®^ (CWR), (2) matching-taper single-cone technique with gutta-percha and AH Plus^®^ (SCGP), and (3) matching-taper single-cone technique with gutta-percha and GuttaFlow^®^ (SCGF). Pre- and postoperative periapical radiographs were performed to detect the presence of endodontic lesions (PAI classification) and to assess the quality of both the obturation and the restoration. Tooth- and patient-related data were collected. Results: The overall endodontic success rate was 75.4% after a mean observation period of 6.3 years. There were no significant correlations between the type or overall quality of obturation and the treatment outcome. Teeth with preoperative lesions had the highest odds ratio (factor of 4.98) for endodontic failure. Tooth- and patient-related variables had no significant effect on endodontic outcome. Conclusions: The preoperative periapical status of teeth requiring endodontic treatment was a substantial prognostic factor for endodontic outcome, whereas the type of obturation material or technique did not affect it.

## 1. Introduction

Successful endodontic treatment plays a critical role in tooth preservation. Over the last decades, there have been major developments in materials and techniques, which have improved the quality of endodontic treatment. However, there is still potential for further improvement [1,2]. The aim of root canal treatment is to completely access the endodontic system, disinfect the dentin, and seal the entire endodontic space. After proper chemo-mechanical preparation, the endodontic system must be adequately sealed to prevent reinfection and promote apical healing [3,4]. For this reason, various types of obturation techniques and materials have been developed.

A root canal filling generally consists of a core material (usually gutta-percha) and a low-viscosity sealing material (sealer), which should both compensate for irregularities in the prepared root canal and seal the dentinal tubules, ramifications, and accessory root canals [5]. The amount of sealer used should ideally be as low as possible, since most sealers are soluble in liquids and dimensional changes occur during their setting [6].

Many studies considered cold lateral compaction as a standard procedure in endodontic obturation [7,8]. It is a cost-effective, predictable, and safe obturation technique with good long-term results [9]. However, cold lateral compaction is also known to be difficult and error prone and is particularly susceptible to the presence of voids, underfilling of curved root canals, and vertical root fractures associated with the application of high compaction forces [10,11,12]. As a simple alternative, matching-taper single-cone obturation was introduced. A tapered gutta-percha master cone corresponding to the final shaping file is used without the need for accessory cones. This method was found to produce results comparable to the cold lateral compaction technique [13,14]. However, since root canals are rarely circular, the spaces adjacent to the prepared round canals are only filled with sealer [15], which may lead to less dense root canal fillings compared to other techniques. In this context, the sealer is of great importance. The characteristics of epoxy-resin-based sealers like AH Plus^®^ (Dentsply Sirona, Charlotte, NC, USA) are reported to represent the benchmark in root filling materials [16,17]. To further simplify root canal filling, a silicone-based non-resorbable sealer (GuttaFlow^®^, Coltène/Whaledent AG, Altstätten, Switzerland) was developed. This sealer is a cold, flowable, self-curing obturation material consisting of gutta-percha particles and a polydimethylsiloxane-based sealer. GuttaFlow^®^ is applied directly into the root canal and then compacted by inserting a gutta-percha master cone. This achieves good material flow into the undercuts and lateral canals but has been associated with a high risk for extrusion of the material into the periapical tissue [18,19]. GuttaFlow^®^ expands slightly during the setting process and, in contrast to AH Plus^®^, is almost insoluble in tissue fluids [20]. Thus, a higher amount of sealer must be applied in order to obtain an adequate obturation and may lead to a higher sealing capacity [21,22]. 

Another development to further improve the sealing of the root canal system was the adhesive root canal filling material Resilon^®^ (Resilon Research LLC, Madison, CT, USA). In accordance with the adhesive technique in restorative dentistry, this synthetic and thermoplastic polymer (polycaprolactone), in combination with a self-conditioning and dual-curing methacrylate-based sealer, was intended to enable an adhesive bond to both the root dentin and the sealer [23]. In theory, this creates a so-called monoblock, which should result in a very dense obturation [24,25]. However, this concept was viewed critically because of the unfavorable geometric shape of root canals and the poor cavity configuration factor (C-factor) [26]. There were suspected severe limitations to adhesive technology regarding material stress and gap formation due to shrinkage processes during and after polymerization and regarding biodegradation of the material induced by bacterial hydrolases [27,28].

Some in vitro studies showed less leakage in root canal fillings with Resilon^®^ and GuttaFlow^®^ compared to lateral condensation of gutta-percha with AH Plus^®^ [29,30]. However, long-term clinical outcome data for root canals filled with the silicone-based sealer GuttaFlow^®^ are lacking. For root-canal-treated teeth obturated with Resilon^®^, two long-term clinical studies have shown a 5.3- to 5.7-fold higher risk of failure compared to those obturated with gutta-percha and AH Plus^®^ [31,32]. An observational study assessing the degraded filling material of non-healed cases requiring retreatment showed that Resilon^®^ was associated with a higher rate of degradation than gutta-percha and sealer [33]. 

The objective of the present study was to determine the success rates of three different endodontic obturation techniques based on a clinical and radiological follow up and to evaluate the effects of various tooth- and patient-related factors on the endodontic outcome. The following obturation materials and techniques were evaluated:

1. Adhesive obturation using the continuous wave of condensation technique with Resilon^®^ (CWR).
2. Matching-taper single-cone obturation with gutta-percha and the epoxy-resin-based sealer AH Plus^®^ (SCGP).
3. Matching-taper single-cone obturation with gutta-percha and the silicon-based sealer GuttaFlow^®^ (SCGF).

The null hypothesis stated that there is no significant difference in treatment outcome between the three root canal obturation techniques evaluated in this study. 

## 2. Materials and Methods

The data for this study were extracted from the dental records of 565 patients with 722 teeth who received endodontic treatment at the Department of Conservative Dentistry and Periodontology at the University of Würzburg (Würzburg, Germany) between 2009 and 2012. Prior approval for this study was obtained from the university’s local ethics committee (approval No. 202/16). Patients with teeth meeting all the inclusion criteria (Table 1) were invited to participate in this study. The exclusion criteria were incomplete records of the patient, not of legal age, not able to perform clinical evaluation, not willing to participate in this study, or deceased. A total of 119 patients, involving 131 teeth, returned for the required clinical and radiologic follow-up examination. The recall rate was 21.1% (119/565). Only one tooth per study participant was included in the statistical analysis to reduce the influence of patient-specific confounding factors. This resulted in a final number of 114 cases with one evaluated tooth per study participant.

All root canal treatments were performed using standardized endodontic procedures including standardized chemo-mechanical preparation and irrigation protocols. The working length was determined using an apex locator (Root ZX, Morita, Saitama, Japan) and verified with a working length radiograph. A working length distance of 0.5 to 1.0 mm from the radiographic apex was preferred. After scouting the root canals and shaping a glide path, the root canal system was prepared with nickel–titanium rotary files (Mtwo^®^, VDW). A typical apical preparation size was ISO 40/.04 in most of the cases. Sodium hypochlorite solution (5%) was used for irrigation in all cases. 

**Figure 1:**
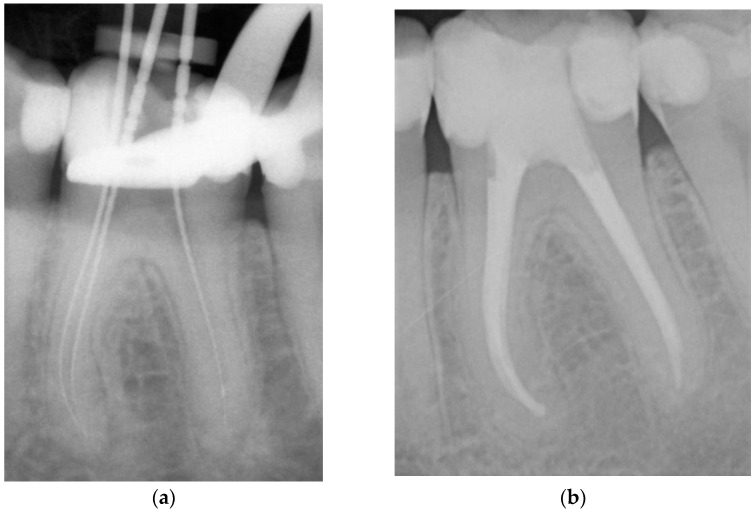
Representative example of root canal filling with Resilon^®^ using the continuous wave of condensation technique (CWR); working length radiograph of tooth 36 (**a**); 6-year follow-up radiograph showing complete apical healing (**b**).

**Figure 2:**
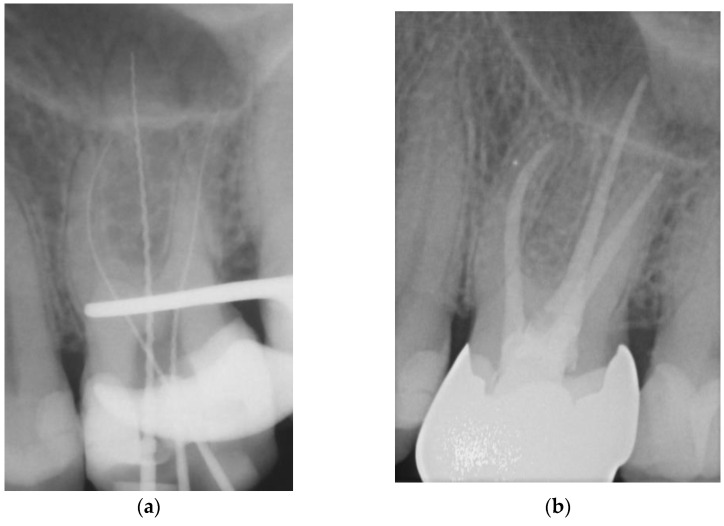
Representative example of root canal filling using the single-cone obturation technique with gutta-percha and AH Plus^®^ (SCGP); working length radiograph of tooth 26 (**a**); 7-year follow-up radiograph showing complete apical healing (**b**).

**Figure 3:**
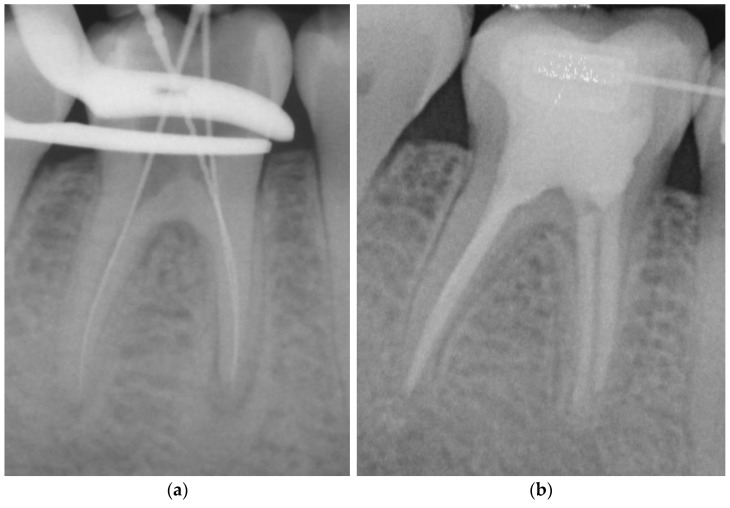
Representative root canal filling using the single-cone obturation technique with gutta-percha and GuttaFlow^®^ (SCGF); working length radiograph of tooth 46 (**a**); 6-year follow-up radiograph showing complete apical healing (**b**).

Root canal obturation was performed using three different obturation techniques:

- Adhesive obturation using the continuous wave of condensation technique with Resilon^®^ (CWR) (Figure 1).
- Matching-taper single-cone obturation with gutta-percha and the epoxy-resin-based sealer AH Plus^®^ (SCGP) (Figure 2).
- Matching-taper single-cone obturation with gutta-percha and the silicon-based sealer GuttaFlow^®^ (SCGF) (Figure 3).

For the adhesive obturation technique, a master cone (RealSeal^TM^, SybronEndo Europe, Amersfoort, The Netherlands) was adjusted to have tug-back 1 mm before working length. The canals were irrigated with an ethylene-diamine-tetra-acetic acid (EDTA) solution and dried with paper points. The primer (RealSeal^TM^, SybronEndo Europe, Amersfoort, The Netherlands) was applied for 30 s. After removing excess material with the paper points, the master cone was thinly coated with the sealer (RealSeal^TM^, SybronEndo Europe, Amersfoort, The Netherlands) and inserted into the root canal. A heat plugger (System B^TM^, Heat Source, SybronEndo Europe, Amersfoort, The Netherlands) was used to create a down-pack of 3 to 5 mm before the working length. The back-fill was performed using heated Resilon^®^-Pellets (RealSeal^TM^, SybronEndo Europe, Amersfoort, The Netherlands) in a gutta-percha gun (Obtura II^TM^, Obtura Spartan Endodontics, Algonquin IL, USA) for a subsequent vertical obturation (continuous wave of condensation technique) of 4 to 5 mm increments.

For single-cone obturation with an epoxy-resin-based sealer (AH Plus^®^, Dentsply Sirona) a matching-taper gutta-percha cone was adjusted to the root canal, having tug-back at the working length. The master cone was thinly coated with AH Plus^®^ and inserted into the root canal.

For single-cone obturation with a silicon-based sealer (GuttaFlow^®^, Coltène/Whaledent GmbH + Co. KG, Langenau) a matching-taper gutta-percha cone was adjusted to the root canal, having tug-back at the working length. GuttaFlow^®^ was injected into the root canal first (adjusted 3 mm before working length), and the master cone wetted with GuttaFlow^®^ was set in afterward.

The teeth were restored with partial or full crowns or regular composite fillings. Radiographic documentation was performed using digital radiographs (VistaScan Mini View, Duerr Dental SE, Bietigheim-Bissing, Germany) or conventional 30 × 40 mm intraoral radiographs (Insight, Kodak). The conventional radiographs were digitized to the highest quality possible for study evaluation.

The follow-up examination was carried out by one of the authors after a mean of 6.26 years (minimum 4.65, maximum 8.68 years). All patients were questioned about their intake of chronic disease medication (CDM, e.g., for diabetes, hypertension, hyperlipidemia), inhalative tobacco consumption in pack years, and the presence of periodontitis. All patients underwent clinical and radiological investigations. The dental status, tooth mobility, and periodontal screening index (PSI) were assessed. Finally, the radiological follow up was performed using digital radiographs (Heliodent^®^ DS, Sirona Dental Systems GmbH, Bensheim, Germany; VistaScan Mini View, Duerr Dental SE, Bietigheim-Bissing, Germany). All radiologic examinations were performed by two senior endodontists with at least ten years of professional experience. After the calibration of both examiners using a set of 100 reference radiographs associated with the Periapical Index (PAI) system, the periapical status of the teeth was assessed using PAI scores [34,35]. The length and homogeneity of root canal fillings and the quality of coronal restorations were also evaluated (Table 2). Teeth with a PAI score of 1 or 2 were classified as successes, and those with a PAI score of 3 or higher were classified as failures [34,35].

Statistical analyses were performed using SPSS^®^ Statistics (IBM, version 25.0). Chi-square tests with Yates continuity correction, Fisher’s exact test, and *t*-tests were applied for the statistical analysis. Phi *φ* and Cramer’s *V* were used as measures of effect size. The odds ratio (OR) was calculated as a measure of the correlation between the ratio of a potential risk factor and the ratio of the occurrence of a disease. The level of significance was set at *α* = 0.05. The inter-rater reliability of evaluating PAI scores at both calibration and data collection was assessed using Cohen’s kappa coefficients [36]. Logistic regression was used to obtain the odds ratio in the presence of more than one explanatory variable.

## 3. Results

A total of 114 teeth from 119 patients with a mean age of 60 years (SD: 15.8 years) were included in this study. The mean recall interval was 6.3 years, and the range was 4.6 to 8.7 years. Before the treatment, 43 teeth had a healthy or sound periapical status (PAI score of 1–2), and 71 teeth had a periapical lesion (PAI score of 3–5). After the treatment, 86 teeth were periapically healthy, and 28 had an apical lesion, yielding an endodontic success rate of 75.4% (Table 3). The inter-rater reliability (kappa coefficients) for the agreement of PAI scores between the two investigators was 0.85 at baseline and 0.95 at the time of control, which can be interpreted as a strong to near complete agreement.

The success rates were 85% for SCGP (34/40) compared to 80% (8/10) for SCGF and 68.7% (44/64) for CWR (Table 4). The chi-square test for independence showed no statistically significant relationship between the success rate and the various obturation techniques ($X^{2}$(2, *n* = 114) = 3.63, *p* = 0.16).

The homogeneity of the obturation (*p* = 0.2) and the extrusion of the root filling material into the periapical tissues (*p* = 0.93) were not related to the selected obturation technique. The length of the root canal filling differed significantly between the groups (*p* = 0.04*). The SCGP technique achieved the highest percentage of adequate root canal fillings compared to SCGF and CWR. The CWR technique showed the highest percentage of underfilled obturations (Table 5).

The endodontic outcome was generally unaffected by the quality of the root canal fillings. The variables obturation length (*p* = 0.12) and homogeneity (*p* = 0.11) as well as the extrusion of root filling material into the periapical region (*p* = 1.00) showed no significant influence on the endodontic outcome. The patient-related variables age (*p* = 0.45), gender (*p* = 0.67), periodontitis (*p* = 0.08), CDM (*p* = 0.19), recall interval (*p* = 0.08), and smoking (*p* = 0.34) showed no significant influence on the endodontic outcome (Table 6). There was no significant difference in success rates between retreatments (70.5%, 31/44) and primary treatments (78.6%, 55/70; *p* = 0.45). In teeth with a preoperative apical lesion, the success rate was 66.2% (47/71) compared to 90.7% (*n* = 39/43) in cases without an apical lesion (*p* = 0.007) (Table 6). The probability of failure in the presence of a preoperative lesion was increased by a factor of 4.98 (OR = 4.98, 95% CI: 1.60, 15.57, *p* = 0.006). With regard to tooth type, molars were more frequently represented with 64 cases than incisors and premolars with 25 cases each. Interestingly, molars had a higher failure rate, but there was no significant correlation between tooth type and endodontic outcome (*p* = 0.07) (Table 6).

## 4. Discussion

The aim of the present study was to evaluate the success rate of endodontic treatment by root canal filling material and technique in a maximally standardized data pool. The null hypothesis stated that there is no significant difference in treatment outcome between the three root canal obturation techniques evaluated in this study (CWR, SCGP, SCGF). The null hypothesis could be proved. At a mean recall interval of 6.3 years, neither the obturation technique nor the obturation materials had a significant effect on the long-term endodontic outcome. The success rate over all groups was 75.4% in a total of 114 cases. Other endodontic outcome studies have reported similar success rates ranging from 68% to 85% [37,38]. The present study indicates that SCGP achieves the highest success rate (85%), followed by SCGF (80%) and CWR (68.7%). However, there was no statistically significant correlation between obturation technique and treatment outcome. A tendency toward a higher rate of non-healing of preoperative periapical lesions was observed in root canals filled with Resilon^®^ (19 of 45 teeth in the CWR group) compared with those filled with gutta-percha and AH Plus^®^ (4 of 21 teeth in the SCGP) or gutta-percha and GuttaFlow^®^ (1 of 5 teeth in the SCGF). A comparable clinical study of 103 root-canal-treated teeth filled with either Resilon^®^ or gutta-percha and AH Plus^®^ showed no significant difference in success rates between the two groups [39]. However, the recall interval was very short (≤24 months). A long-term observational study with a mean follow up of 5.8 years concluded that Resilon^®^ achieved a success rate of only 56% compared to 88% with gutta-percha and AH Plus^®^ [31]. Resilon^®^ had a significantly higher probability of endodontic failure compared with gutta-percha (OR = 5.7). A clinical study of 125 teeth also showed lower endodontic success rates after adhesive obturation with Resilon^®^ [32]. Given the 5.3-fold higher probability of an endodontic lesion after root canal treatment with Resilon^®^, it was concluded that Resilon^®^ may lead to a worse long-term endodontic outcome than gutta-percha and AH Plus^®^. The mean follow-up interval (12.4 years for Resilon^®^ fillings and 12.1 years for gutta-percha fillings) was significantly longer than in the present study (6.3 years). The authors discussed that the high risk of bacterially induced biodegradation of the obturation material may contribute to the higher failure rate of Resilon^®^ [40]. The observation period in the present study may have been too short to demonstrate a negative impact of the biodegradation of the Resilon^®^ material. 

In the present study, the group of root-canal-treated teeth obturated with gutta-percha and the silicone-based sealer GuttaFlow^®^ (SCGF) contained a very small number of cases (*n* = 10). The success rate of SCGF appeared to be comparable to that of the other two techniques. Numerous in vitro studies on the physicochemical properties, marginal adaptation characteristics, and bacterial leakage resistance of GuttaFlow^®^ suggest that this material is associated with a clinically promising endodontic outcome [18,21,41,42,43]. However, comprehensive clinical studies quantifying the success rates of root canal treatment with GuttaFlow^®^ are currently lacking. Therefore, randomized controlled trials are needed.

All primary endodontic treatments and retreatments were performed by four experienced endodontists with at least three years of training in endodontics. In addition, all operators worked at our teaching hospital and followed standardized chemo-mechanical preparation and irrigation protocols during all root canal treatments.

In this study, conventional root filling materials were investigated. Modern endodontic materials such as bioceramic or bioactive sealers have been introduced recently. However, to date, their supposed more favorable results in periapical healing have low evidential support [44].

A severe limitation of the present study is the low recall rate of 21.1%. Several reasons must be considered for not recording 446 of 565 root-canal-treated teeth in the defined observation period of four years. There was incomplete documentation in 34.0% of the cases, no response from the patient in 19.5% of the cases, evident tooth extraction in 11.0% of the cases, and various other reasons in 14.5% of the cases. However, great efforts were made to keep the sample size for recall as large as possible, in particular by carefully reviewing all data available at the dental clinic. Most of the included patients attend the clinic for general dental care. Almost half of the patients with missing recall data did not meet the well-defined inclusion criteria, did not have proper clinical and radiographic diagnostics (192 of 565 patients), or had undergone tooth extraction for prosthetic reasons (62 of 565 patients). The remaining drop-outs were patients who could not be reached (110 of 565 patients), declined to participate (56 of 565 patients), did not appear for this study (14 of 565 patients), were not of legal age (7 of 565 patients), or were deceased (5 of 565 patients).

Assessment of the technical quality criteria revealed that the studied root canal filling groups differed significantly in terms of filling length. The percentage of underfilled canals was significantly higher with CWR compared with SCGP or SCGF. In contrast, another clinical study found no difference in radiographic obturation length between the continuous wave of condensation technique (*n* = 180) and the single-cone technique (n = 160) [45]. The ability of CWR to achieve an adequate length of root filling may be limited by the need to adjust the master point according to the verified working length. In the CWR group, the tip of the gutta-percha point was generally set 1 mm short of the working length to prevent extrusion of the root filling material during the vertical compaction phase. It was reported that the vertical obturation technique with warm gutta-percha may be associated with an increased incidence of overextension [46]. In the present study, there were no significant differences in the extrusion of root filling material between the groups, and the success rate of endodontic treatment was not significantly affected by any of the quality criteria (length, homogeneity, extrusion of RCF). However, a correlation between the length of the root canal filling and the endodontic outcome has been observed in other studies [3,47]. Consistent with numerous studies, we found that the preoperative periapical condition was the most important prognostic factor (OR = 4.98) for the success or failure of root canal treatment [3,47]. Within the limitations of this retrospective study, tooth- and patient-related variables (the so-called secondary outcome factors) can be considered as having minor relevance and effect on the endodontic outcome. In accordance with the clinical study of Jahreis, et al. [48], there was no significant correlation between CDM and the endodontic outcome, at least highlighted in a population of 60 years [48]. However, the results of a prospective study indicate that several patient- and tooth-related variables (e.g., diabetes, pathological periodontal probing depths, or lower molars in terms of tooth type) may be associated with a higher risk for tooth loss or endodontic failure [49].

## 5. Conclusions

Within the limitations of this study, it can be concluded that the preoperative periapical status of teeth requiring endodontic treatment is a substantial prognostic factor for endodontic outcome, whereas the type of obturation material or technique did not affect endodontic outcome. 

## Figures and Tables

**Table 1:** Inclusion criteria: A complete data set containing the following information was required for inclusion in this study.

Records data	Anamnesis, type and progress of endodontic treatment, obturation material and technique, periapical radiographs
Radiographic data	Preoperative, working length, and postoperative radiographs of high quality using a digital imaging assessment (VistaScan Mini View, Dürr Dental SE) with a high resolution of 22 line pairs each millimeter
Demographic data	Age and gender of the participants in this study at the time of the follow-up examination
Time of treatment, operator	Endodontic treatment at the University of Würzburg, Department of Conservative Dentistry (Würzburg, Germany), between 2009 and 2012
Obturation technique	- Adhesive obturation using the continuous wave of condensation technique with Resilon^®^ - Single-cone obturation with gutta-percha and the epoxy-resin-based sealer AH Plus^®^ - Single-cone obturation with gutta-percha and the silicone-based sealer GuttaFlow^®^

**Table 2:** Quality criteria for the assessment of the root canal filling (RCF) [11,34].

PAI	1	Sound periapical status
	2	Minor changes in bone structure
	3	Changes in bone structure with mineral loss
	4	Apical periodontitis with defined lesion
	5	Severe apical periodontitis with signs of exacerbation
Length of RCF	Adequate	RCF ending ≤ 2 mm from the radiographic apex
	Overfilled	RCF ending beyond the radiographic apex
	Underfilled	RCF ending > 2 mm from the radiographic apex
Homogeneity of RCF	Homogeneous	Absence of voids within or between fillings and root canal walls
	Inhomogeneous	Presence of voids within or between fillings and root canal walls

**Table 3:** Periapical status of root-canal-treated teeth before and after treatment.

	Total [*n*]	Sound [*n*] (%)	Diseased [*n*] (%)
Before intervention	114	43 (37.7%)	71 (62.3%)
After intervention	114	86 (75.4%)	28 (24.6%)

*n* = sample size.

**Table 4:** Success and failure rates by obturation technique.

Obturation Technique	Total [*n*] (%)	Success [*n*] (%)	Failure [*n*] (%)
CWR	64 (56.1%)	44 (68.7%)	20 (31.3%)
SCGP	40 (35.1%)	34 (85.0%)	6 (15.0%)
SCGF	10 (8.8%)	8 (80.0%)	2 (20.0%)
Total [*n*]	114 (100%)	86 (75.4%)	28 (24.6%)

*n* = sample size.

**Table 5:** Quality of RCF depending on the obturation technique.

Quality Criteria		CWR (*n* = 64)	SCGP (*n* = 40)	SCGF (*n* = 10)	*p*	*Effect Size*
Homogeneity	Homogeneous	51 (79.7%)	27 (67.5%)	9 (90.0%)	0.20	
	Inhomogeneous	13 (20.3%)	13 (32.5%)	1 (10.0%)		
Extrusion of RCF	No extrusion	54 (84.4%)	33 (82.5%)	8 (80.0%)	0.93	
	Extrusion	10 (15.6%)	7 (17.5%)	2 (20.0%)		
Length of RCF	Adequate	46 (71.9%)	37 (92.5%)	8 (80.0%)	0.04 *	0.24 *(Cramer’s V)*
	Underfilled	12 (18.7%)	3 (7.5%)	0 (0.0%)		
	Overfilled	6 (9.4%)	0 (0.0%)	2 (20.0%)		

*n* = sample size; *p* = significance value using chi-square test; * = significant, *p* ≤ 0.05.

**Table 6:** Endodontic outcome by tooth- and patient-related variables.

		Total [*n*] (%)	Success [*n*] (%)	Failure [*n*] (%)	*p*	Effect Size
Preoperative periapical status	Sound	43 (37.7%)	39 (90.7%)	4 (9.3%)	0.007 *	0.28 *(Phi)*
	Diseased	71 (62.3%)	47 (66.2%)	24 (33.8%)		
	Total	114 (100%)	86 (75.4%)	28 (24.6%)		
Type of treatment	Primary treatment	70 (61.4%)	55 (78.6%)	15 (21.4%)	0.45	
	Retreatment	44 (38.6%)	31 (70.4%)	13 (29.6%)		
	Total	114 (100%)	86 (75.4%)	28 (24.6%)		
Periodontitis	Present	81 (73.0%)	58 (71.6%)	23 (28.4%)	0.08	
	Absent	30 (27.0%)	27 (90.0%)	3 (10.0%)		
	Total	111 (100%)	85 (76.6%)	26 (23.4%)		
Quality of coronal restoration	Adequate	107 (93.9%)	82 (76.6%)	25 (23.3%)	0.36	
	Inadequate	7 (6.1%)	4 (57.1%)	3 (42.9%)		
	Total	114 (100%)	86 (75.4%)	28 (24.6%)		
Tooth type	Incisors	25 (21.9%)	23 (92.0%)	2 (8.0%)	0.07	
	Premolars	25 (21.9%)	19 (76.0%)	6 (24.0%)		
	Molars	64 (56.2%)	44 (68.7%)	20 (31.3%)		
Gender	Male	45 (39.5%)	33 (73.3%)	12 (26.7%)	0.67	
	Female	69 (60.5%)	53 (76.8%)	16 (23.2%)		
	Total	114 (100%)	86 (75.4%)	28 (24.6%)		
Chronic disease medication	Present	47 (42.3%)	39 (83.0%)	8 (17.0%)	0.19	
	Absent	64 (57.7%)	45 (70.3%)	19 (29.7%)		
	Total	111 (100%)	84 (75.7%)	27 (24.3%)		
Smoking	Present	27 (23.7%)	18 (66.7%)	9 (33.3%)	0.34	
	Absent	87 (76.3%)	68 (78.2%)	19 (21.8%)		
	Total	114 (100%)	86 (75.4%)	28 (24.6%)		

*n* = sample size; *p* = significance value using chi-square test; * = significant, *p* ≤ 0.05.

## Data Availability

Data are available in a publicly accessible repository. The data presented in this study are openly available at OPUS Würzburg (Dissertation of the Faculty of Medicine/Department of Conservative Dentistry and Periodontology; DOI: https://doi.org/10.25972/OPUS-27815; URN: urn:nbn:de:bvb:20-opus-278158).
